# Dissociation of extrastriate body and biological-motion selective areas by manipulation of visual-motor congruency

**DOI:** 10.1016/j.neuropsychologia.2009.07.012

**Published:** 2009-12

**Authors:** Ioannis Kontaris, Alison J. Wiggett, Paul E. Downing

**Affiliations:** Wales Institute of Cognitive Neuroscience, School of Psychology, Bangor University, Bangor, United Kingdom

**Keywords:** Action perception, Extrastriate body area, Fusiform body area, Superior temporal sulcus, fMRI, Visuo-motor control, Biological motion

## Abstract

To date, several posterior brain regions have been identified that play a role in the visual perception of other people and their movements. The aim of the present study is to understand how these areas may be involved in relating body movements to their visual consequences. We used fMRI to examine the extrastriate body area (EBA), the fusiform body area (FBA), and an area in the posterior superior temporal sulcus (pSTS) that responds to patterns of human biological motion. Each area was localized in individual participants with independent scans. In the main experiment, participants performed and/or viewed simple, intransitive hand actions while in the scanner. An MR-compatible camera with a near-egocentric view of the participant's hand was used to manipulate the relationship between motor output and the visual stimulus. Participants’ only view of their hands was via this camera. In the Compatible condition, participants viewed their own live hand movements projected onto the screen. In the Incompatible condition, participants viewed actions that were different from the actions they were executing. In pSTS, the BOLD response in the Incompatible condition was significantly higher than in the Compatible condition. Further, the response in the Compatible condition was below baseline, and no greater than that found in a control condition in which hand actions were performed without any visual input. This indicates a strong suppression in pSTS of the response to the visual stimulus that arises from one's own actions. In contrast, in EBA and FBA, we found a large but equivalent response to the Compatible and Incompatible conditions, and this response was the same as that elicited in a control condition in which hand actions were viewed passively, with no concurrent motor task. These findings indicate that, in contrast to pSTS, EBA and FBA are decoupled from motor systems. Instead we propose that their role is limited to perceptual analysis of body-related visual input.

## 1 Introduction

**Fig. 1:**
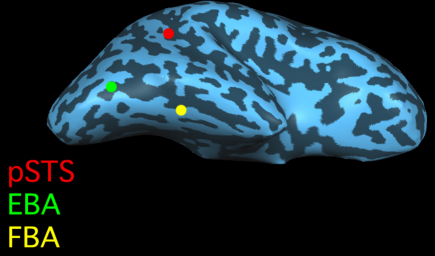
Schematic illustration of key brain areas involved in the perception of human bodies, body parts, and bodily movements. The extrastriate body area (EBA) is located in the inferior temporal sulcus, and the fusiform body area (FBA) in the lateral fusiform gyrus. Both regions respond strongly and selectively to static and dynamic images of human bodies and body parts. A region in the posterior superior temporal sulcus (pSTS) responds preferentially to human biological motion.

Recent studies of monkeys and humans have identified several regions of the inferior and lateral occipitotemporal cortex that respond selectively to visually presented bodies, body parts, and/or patterns of biological movement (Fig. 1). Among these areas is the superior temporal sulcus (STS), where single-unit studies in monkeys reveal cells that respond to visually perceived biological actions, and where human and monkey fMRI shows patches that are activated by images of bodies or body movements (Allison, Puce, & McCarthy, 2000; Pinsk, DeSimone, Moore, Gross, & Kastner, 2005; Pinsk et al., 2009; Puce & Perrett, 2003). Furthermore, in humans, fMRI reveals two regions – the extrastriate body area (EBA) in the inferior temporal sulcus and the fusiform body area (FBA) in the lateral fusiform gyrus – that respond strongly and selectively to static and dynamic images of human bodies and body parts (Downing, Chan, Peelen, Dodds, & Kanwisher, 2006a; Downing, Jiang, Shuman, & Kanwisher, 2001; Peelen & Downing, 2005a; Schwarzlose, Baker, & Kanwisher, 2005). In the case of EBA, multiple sources of converging evidence support the hypothesis that this area performs a visual analysis of the appearance of the body (Peelen & Downing, 2007). Less evidence is extant concerning FBA, where efforts have mainly focused on distinguishing this region from the fusiform face area, which it overlaps (Schwarzlose et al., 2005).

Are these brain areas strictly visual, or is their activity modulated by motor behaviour? More specifically, are they involved in relating visual input to motor output? Several lines of relevant evidence are found in the literature. First, with respect to STS, there is some evidence from single-unit studies that neural activity is reduced for self-generated movements. For example, Perrett and colleagues (Hietanen & Perrett, 1993; Perrett, Mistlin, Harries, & Chitty, 1990) have reported cells in the upper bank of the STS for which the experimenter's hand movements generated a large response, but similar movements by the animal did not produce a response greater than baseline. More recently, a human fMRI study provided related evidence: The introduction of small delays into a live video of participants’ own hand movements increased activity in the vicinity of posterior STS, in direct proportion to the length of the delay (Leube et al., 2003). So STS, or at least a posterior sub-region of STS, appears to be modulated by the relationship between the visual input and motor activity, perhaps (in part) to suppress the perceptual consequences of one's own actions in order to preferentially represent others’ actions. This account is consistent with the proposal that STS is generally involved in the understanding of other individuals’ behaviour and mental states (Allison et al., 2000; Puce & Perrett, 2003). (Note however that the possible homology between the regions of macaque and human STS discussed here has not been established.)

Some recent fMRI studies indicate motor modulation of the responses in EBA. For example, Jackson, Meltzoff, and Decety (2006) reported increased activity in a region consistent with EBA for the imitation of actions, relative to the passive viewing of the same actions—although these conditions differ in their attentional demands, which may account for this effect. Astafiev, Stanley, Shulman, and Corbetta (2004) reported activity in an area overlapping with EBA that was elicited by the preparation of visually guided (but unseen) movements of the hand or foot. These findings have been interpreted as evidence that EBA receives not only visual input but also input related to motor commands—and further that it might meet criteria for a system that can provide signals distinguishing one's own body parts and movements from those of other people (Jeannerod, 2004; see also David et al., 2007; David et al., 2009—considered at length in Section 4). This interpretation was challenged by Peelen and Downing (2005b), who used multivoxel pattern analyses to show that the lateral occipitotemporal regions activated by visually presented bodies and by unseen finger movements were distinct. However, given that the procedures used by Peelen and Downing (2005b) and by Astafiev et al. (2004) differ somewhat, this debate has not yet been resolved.

The aim of the present study was to provide a novel and combined test of the role of STS, EBA, and FBA in visuo-motor function. We compared situations in which the visual stimulus and the participants’ motor output were systematically varied. In one condition, participants viewed live video images of their own hands performing simple intransitive actions. In a second condition, a recording of the same visual stimulus was presented, while participants performed hand actions in a different sequence. Thus in both conditions, hand movements were performed and hand movements were viewed; in one condition these two were compatible, while in the other they were incompatible. Additional control conditions involved performing hand movements without visual input, and seeing hand movements with no concurrent motor task. The responses to these four conditions were assessed in EBA, FBA, and a region of posterior STS, each localized in individual participants with independent fMRI contrasts. To the extent these areas can be considered “visuo-motor”, we would expect to see modulation of their responses as a function of the relationship between the visual stimulus and the motor output. Alternatively, a strictly visual area would respond according to the visual stimulus, independent of concurrent motor output.

## 2 Methods

### 2.1 Participants

Eleven healthy volunteers (9 female, 2 left-handed, mean age 24 years) were recruited from the Bangor University community. Participants satisfied all requirements in volunteer screening and gave informed consent. Procedures were approved by the Ethics Committee of the School of Psychology at Bangor University. Participation was compensated at £15 per session.

### 2.2 Design and procedure

#### 2.2.1 Main experiment

The stimuli were movies of the participants’ own hands performing the actions, recorded during the experiment with an MR-compatible analogue video camera. The actions consisted of repeated series of transitions from one gesture to another. Three gestures were used: (a) closed fist, apart from the thumb pointing upward, (b) an open hand with the palm down and (c) extended index and little finger with the tip of the thumb in contact with the withdrawn middle and ring finger. The actions consisted of these gestures performed repeatedly in succession, in all possible orders (ABC, ACB, BAC, BCA, CAB, CBA). Participants were trained to perform these hand actions in response to auditory cues (for example “A, B, C”). The association of auditory letter cues to each gesture was counterbalanced across participants. Participants completed a training phase outside the MRI scanner before participating in the experiment.

**Fig. 2:**
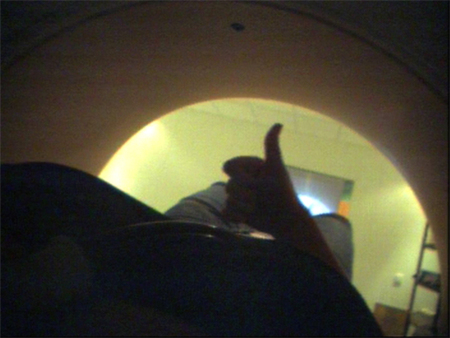
Frame capture of a participant's view from the MR-compatible camera.

The participants lay in the scanner in a supine position, with the head slightly raised above the body axis. They wore earphones and viewed a projection screen, via a mirror, placed at the foot of the scanner bed. The MR-compatible video camera was attached to the head coil, aligned so that the participant's right arm and hand were visible, approximating an egocentric view (see Fig. 2). The hand was positioned alongside, but not touching, the body. A hand rest was used in order to minimize movements of the hand. Before scanning we confirmed that the participants’ hands were not visible to them directly but only via the projection screen.

**Fig. 3:**
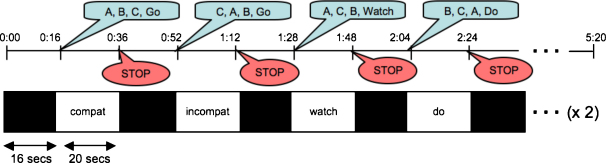
Design of the experimental task. In a blocked design, each 20 s experimental block began with auditory instructions signalling the order in which the hand gestures were to be performed plus the task instruction (e.g. “B, A, C, Go”). The three gestures were performed at a frequency of approximately one action per second, in a repeating cycle, until the end of the block (signalled by an auditory “Stop” instruction). After each experimental block, participants closed their eyes for 16 s.

The design of the experiment is illustrated in Fig. 3. There were four experimental conditions (Compatible, Incompatible, Watch, and Do). In each run these conditions occurred twice, in that order. In the Compatible condition, participants viewed their own hand movements projected onto the screen—hence the visual input was congruent with the current motor output. In the Incompatible condition, participants viewed their own actions that had been recorded during the immediately preceding block of the same scan, while executing actions in a different, and hence incongruent, sequence. In the Watch condition, the same actions were viewed again, this time passively. Finally, in the Do condition, participants performed the actions in a new sequence, without any concurrent visual input.

In the first 4 s of every 20-s experimental block, three cues were read out to the participant, followed by a task instruction (e.g. “B, A, C, Go”) that would indicate the action order and the required task. A “Watch” instruction cued the participant to watch the actions without moving; a “Do” instruction cued performance of the gestures without visual input; and a “Go” instruction cued performance of the actions while simultaneously attending the visual stimulus. The gestures were performed (or viewed, in the Watch conditions), at a frequency of approximately one action per second, in a repeating cycle, until the end of the block (signalled by an auditory “Stop” instruction). Simultaneous with the task instruction, the visual signal (depending on condition) would commence. For example, at this point in a Compatible block, the live video view of the participant's hand in motion would become visible; in an Incompatible or Watch block, the recorded movements from the immediately preceding Compatible block would appear. Participants were instructed to close their eyes at the beginning of each scan, and between blocks, as indicated by the “Stop” instruction; and to open them upon hearing a task instruction.

Recording of movements during the Compatible blocks was achieved by splitting the output of the MR-compatible video camera so that the same signal could be displayed live via the analog input of the data projector, and simultaneously digitised and recorded via computer software. In each scan, which lasted 5 min 20 s, the four conditions were tested twice (Fig. 2). Each participant performed 4 scans, yielding 8 blocks per participant per condition.

#### 2.2.2 Localizers

Each participant was scanned on a series of blocked-design experiments. These were used to localize a priori regions-of-interest (ROIs) separately for each subject. To localize body-selective EBA and FBA, images of human bodies (without heads), and images of chairs were presented in separate blocks. Each run comprised 21 blocks of 16 s duration each. Five of these blocks (1, 6, 11, 16 and 21) were fixation-only baseline conditions. Throughout the remaining 16 blocks, participants observed stimuli from one of the four conditions. Twenty stimuli were presented per block (300 ms on/450 ms off). Twice during each stimulus block, the same image was presented two times in succession. Participants were instructed to detect these immediate repetitions and report them with a button press (1-back task). The position of each stimulus image varied slightly on alternate presentations, in order to prevent performance of the 1-back task based on low-level visual transients. The order of the blocks was symmetrically counterbalanced within each run. Participants completed two runs of this localizer task.

In order to localize the region of posterior STS that responds to whole-body human movements, biological-motion stimuli in the form of point-light displays were contrasted with scrambled motion sequences of the same animation stimuli (Shipley & Brumberg, n.d.). The stimuli depicted a variety of whole-body movements. One scan was carried out for each participant. Each point-light animation lasted 1 s at 30 frames per second, followed by a 1 s fixation. The 12 dots defining each point-light stimulus were white against a black background. Scrambled motion sequences were produced from the exact same motion vectors found in the biological animations, but the starting position of each dot was randomized and controlled so as to keep the density of the stimulus constant and comparable to the original biological-motion stimuli in terms of local motion. Participants performed the “1-back” task during these scans.

### 2.3 Data acquisition

All imaging data were obtained using a 3T Philips Achieva MRI scanner, equipped with a SENSE parallel head coil (*Philips*, Best, Netherlands). Stimuli were presented using a Sanyo LCD projector (*Sanyo*, Osaka, Japan) directed at a rear-projection screen and was administered using MatLab (*The MathWorks*, Natick, MA) and Psychophysics Toolbox (Brainard, 1997; Pelli, 1997), running on an Apple Mac Pro computer. Hand actions were recorded using an MRI-compatible video camera (*MRC Systems*, Heidelberg, Germany) and their play-back projection was delivered with iMovie (*Apple*, Cupertino, CA). Participants’ responses on the 1-back task were recorded using a nonferrous, fiber-optic response keypad (*Current Designs*, Philadelphia, PA).

Functional data acquisition was achieved with T2*-weighted scans using a single-shot echo planar (EPI) sequence. Acquisition parameters for all participants were: 34 off-axial slices, 64 × 64 matrix, slice thickness = 3 mm, voxel dimensions = 3 × 3 mm in-plane; echo time (TE) = 50 ms; repetition time (TR) = 2000 ms; flip angle = 90°. Coverage included the entire cortex and most of the cerebellum. Parameters for T1-weighted scans, which served as an anatomical reference for each participant, were: 256 × 256 matrix; slice thickness = 1.3 mm; voxel dimensions = 1 mm × 1 mm in-plane; TR = 16 ms, TE = 3 ms; flip angle = 8°.

### 2.4 Data analyses

Preprocessing and statistical analyses of the MRI data were performed using BrainVoyager QX 1.9 (*Brain Innovation*, Maastricht, The Netherlands). Seven dummy volumes were obtained prior to each scan in order to minimize T1 saturation effects. Functional data were motion corrected, and low-frequency drifts removed with a temporal high-pass filter (0.006 Hz). No spatial smoothing was applied. Functional data were manually co-registered with the three-dimensional anatomical T1 scans. The three-dimensional anatomical scans were transformed into Talairach space (Talairach & Tournoux, 1988), and the parameters from this transformation were subsequently applied to the co-registered functional data which were re-sampled to 1 mm × 1 mm × 1 mm voxels.

For each participant, general linear models (GLMs) were created for the localizers and the main experiment. A boxcar predictor was convolved with a 2γ hemodynamic response function (HRF) for each stimulus condition. Regressors of no interest were also included to control for across-scan differences in the mean MR signal. Regressors were fitted to the MR time-series in each voxel and the resulting parameter (beta) estimates were used to estimate the response magnitude for each condition of interest.

The ROI analyses were focused on the right hemisphere, since findings from prior research indicate a limited or absent left hemisphere manifestation of FBA and pSTS (Grossman et al., 2000; Peelen & Downing, 2005a). For each ROI (EBA, FBA, pSTS) in each subject, the most significantly activated voxel was identified within a restricted part of cortex based on previously reported anatomical locations (EBA: Downing, Wiggett, & Peelen, 2007; FBA: Peelen & Downing, 2005a; pSTS: Grossman et al., 2000; Downing, Peelen, Wiggett, & Tew, 2006b). ROIs were defined as the set of contiguous voxels that were significantly activated (all *p* < 0.0001 uncorrected, *p* < 0.005 for STS) within a 10 mm cube surrounding the peak voxel. Within each ROI in each subject, a further GLM was applied modelling the aggregate response of the voxels in the region to the experimental conditions of the main experiment. The beta values from these regression analyses provided estimates of the response to the experimental conditions within each ROI, which were subsequently analyzed with ANOVAs.

We also computed percent signal change timecourse plots for data from the ROIs. These were created by expressing the mean BOLD response, averaged across voxels within each ROI for each scan in each participant separately, in terms of percent signal change from the baseline time-points (during which participants’ eyes were closed). Time-locked averages were then accumulated for each condition, across scans and participants.

Finally, we performed a whole-brain, random-effects analysis contrasting the response to the Compatible and Incompatible conditions. The uncorrected voxelwise threshold was set at *p* < 0.005. Using the cluster-size threshold plug-in for BrainVoyager QX, Monte Carlo simulations showed that for cluster size > 6 acquired voxels, the effective corrected threshold is *p* < 0.005.

## 3 Results

Two scans from the main experiment (from different participants) were discarded due to experimental errors. For one participant, only one EBA/FBA localizer scan was conducted.

The ROIs were successfully localized in all participants (although it was necessary to reduce the threshold to *p* < 0.05 in three participants in order to identify a pSTS region of interest). The mean sizes (with SEM) of the ROIs were: EBA: 707 mm^3^ (41), FBA: 379 mm^3^ (70), pSTS: 350 mm^3^ (93). The average Talairach coordinates (with SEM) of the ROI peak voxels were: EBA: 46.6 (1.4), −63 (2.5), −6.7 (2.5), FBA: 40.5 (1.6), −41.9 (0.8), −22.6 (1.7), pSTS: 51.8 (2.4), −47.9 (1.5), 8.4 (1.9).

**Fig. 4:**
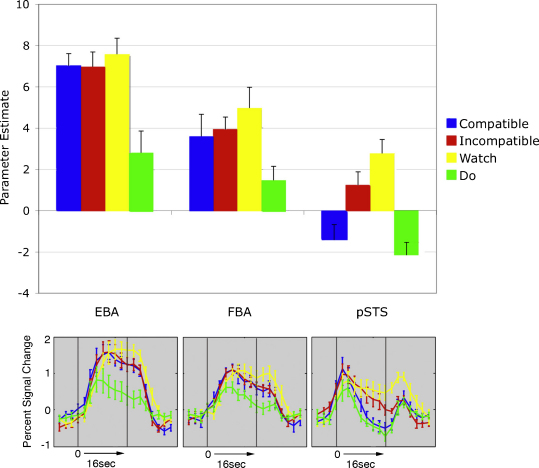
Results of the region of interest analysis. The top panel shows mean regression parameter estimates for the response to each experimental condition (Compatible, Incompatible, Do, Watch), in the right hemisphere extrastriate body area (EBA), fusiform body area (FBA), and posterior superior temporal sulcus (pSTS). Error bars show within-subjects standard error of the mean calculated for each ROI separately. The bottom panel shows the time course of activation in each of the ROIs. Percent signal change is shown relative to the baseline (eyes-closed) condition. The two vertical grey lines indicate the start and end of the experimental blocks (16 s).

Initially, a repeated-measures 3 × 4 within-participants ANOVA with factors ROI (pSTS, EBA, FBA) and condition (Compatible, Incompatible, Watch, Do) revealed a significant interaction between ROI and condition, *F*(6, 60) = 6.0, *p <* 0.001 (Fig. 4). Follow up analyses tested the effects of condition in each ROI individually. In each case, a one-way ANOVA showed a significant modulation of the BOLD response by experimental condition: EBA, *F*(3, 30) = 34.0, *p <* .001; FBA, *F* (3, 30) = 9.8, *p* < .001; pSTS, *F*(3, 30) = 18.3, *p <* .001.

Further comparisons explored the effects of interest in each area. To assess the effects of congruency between the visual stimulus and motor output when both were present, we compared the BOLD responses in the Compatible and Incompatible conditions. Only in pSTS was this difference significant, *t*(10) = 4.0, *p* < .005 (rEBA: *t*(10) = 0.18; rFBA: *t*(10) = 0.52).

To assess whether motor output *per se* modulated the response of these areas to identical visual images of moving hands, we compared the Watch and the Incompatible conditions in each area. Only in pSTS was this difference significant, *t*(10) = 3.4, *p* < .01 (rEBA: *t*(10) = 1.7; rFBA: *t*(10) = 1.6). A similar result was obtained when comparing the Compatible, Incompatible, and Watch conditions (all of which consisted of identical visual input, but varying in the presence and nature of motor output). A one-way ANOVA for each area produced a significant main effect for pSTS, *F*(2,20) = 21.1, *p* < 0.001, but no significant effects for rEBA, *F*(2,20) = 1.9, *p* = 0.17, nor for rFBA, *F*(2, 20) = 2.8, *p* = 0.09.

In a post hoc analysis to test whether viewing self-generated actions activates pSTS at all, relative to performing actions alone, we compared the response in the Compatible condition (congruent visual stimulus and motor output) with the Do condition (motor output only). This difference was not significant, *t*(10) = 1.3, *p* = 0.22.

The same pattern of results was found in all of the ROI analyses when the data from the two left-handed participants were excluded.

**Fig. 5:**
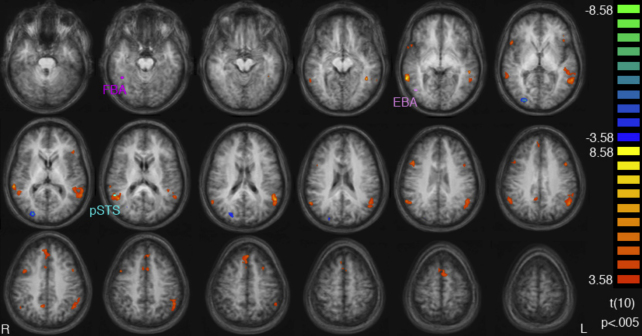
Activations from the whole-brain, random-effects group average analysis (overlaid on the average anatomical scan from all participants). Axial slices are shown in 5 mm increments starting at *z* = −25 (top left) to *z* = 60 (bottom right). Activations are thresholded at *p* < 0.005, cluster-size threshold: 6 voxels. The regions shown in orange are those that responded more in the Incompatible than in the Compatible condition. The regions shown in blue were more active for the reverse contrast. See Table 1 for details. For comparison, the approximate locations of each ROI peak voxel (the average Talairach coordinates from the individual subject ROI analyses) are also marked.

In the whole-brain analysis (Fig. 5), the contrast incompatible > compatible produced significant activations in several brain areas (listed in detail in Table 1). These were mainly clustered around left and right superior temporal/inferior parietal cortex, the medial prefrontal wall, and the lateral prefrontal cortex. Furthermore, there was a significant activation for compatible > incompatible in parieto-occipital cortex.

Finally, we performed a post hoc, whole-brain contrast of Do > baseline, in order to test whether the positive response in EBA and FBA to the Do condition was specific to these regions. When the threshold was lowered sufficiently so that the whole-brain activations in this contrast began to include EBA and FBA, most of the visual cortex was also active. This indicates that the positive response to the Do condition in these areas reflects a non-specific response to the visual percept of the experimental environment, compared to the eyes-closed baseline periods.

## 4 Discussion

Our results provide a clear contrast among regions of the occipitotemporal cortex that respond to images of bodies, body parts, and their movements. In the Compatible and Incompatible conditions, the visual input was identical, but in one case this corresponded to the participant's current movements, while in the other it did not. Right hemisphere EBA and FBA responded strongly in these conditions, but did not distinguish between them. Nor did the response in these conditions differ from the condition in which participants passively viewed hand actions without any concurrent motor output. This pattern of findings is most parsimoniously consistent with a model in which EBA and FBA are not involved in computing relationships between visual input and motor output, but instead play a basic role in analysing body-related visual input (Peelen & Downing, 2007).

The present results run counter to a recent finding suggesting that EBA plays a role in interpreting whether visual stimuli are self- or other-generated (David et al., 2007). Participants used a joystick to guide a cursor to one of two visual targets. On congruent trials, the cursor followed the participants’ movements accurately, while on incongruent trials, the path of the cursor deviated slightly from the true path generated by the participant. Right EBA showed a greater response to incongruent than congruent trials. However, many other brain areas showed the same pattern, indicating possible attentional differences between the conditions.

A further possibility is that the stimuli used by David et al. (2007) engaged motion-sensitive area MT rather than EBA (considering that these areas overlap (Downing et al., 2007), and that the stimuli were moving cursors rather than body parts). This is consistent, for example, with a TMS study of motion-selective area MT (Whitney et al., 2007), which concluded that MT plays a role in interpreting and compensating for self-generated movements in goal-directed reaching. David and colleagues addressed this possibility in a later study using TMS (David et al., 2009), showing that stimulation of left EBA,^1^ compared to a control site, slowed participants’ detection of movements that did not correspond to their current motor output. They argued against a role for MT in this effect, on the grounds that their left EBA stimulation did not affect accuracy on a motion discrimination task. However, examination of the data (Table 2 of David et al., 2009), suggests there may have been a performance effect. In d-prime terms, EBA (and/or MT) stimulation resulted in lower accuracy on the motion task than did stimulation of the control site; and furthermore the former, relative to the latter, appears to have prevented learning on the task: EBA/MT pre-TMS: 0.87; post-TMS: 0.86; control site pre-TMS: 1.02; control post-TMS: 1.31.

The current results also support the claim that EBA activity is not modulated by the performance of unseen hand movements (Peelen & Downing, 2005b). Additional relevant evidence is found in the whole-brain contrast of Incompatible vs Compatible, where we identified a region ∼1.5 cm anterior and lateral to the right EBA. (This region was not selective to either bodies or to point-light biological motions, as measured with localizer data; both *p* > 0.50.) Peelen and Downing (2005b), following the procedure of Astafiev et al. (2004), identified a similar region ∼1 cm anterior to their localized EBA that was more responsive to unseen visually guided finger movements compared to a visually matched baseline. Based on this finding, the apparent modulation of EBA by the preparation to make an unseen movement (Astafiev et al., 2004) may instead be attributable to MST, which responds to tactile as well as visual stimulation (Beauchamp, Yasar, Kishan, & Ro, 2007). We speculate that the middle temporal region identified here could match that found by Peelen and Downing (2005b), and in the present study could reflect increased attention to somatosensory representations when these conflict with visual input. Alternatively, this region may serve a more general attentional function; a similar region has been identified in a study of spatial attention (Gitelman et al., 1999) and as a possible part of the “multiple demands” attentional network (Duncan, 2006).

Considering the above evidence together, additional experiments will be needed to clarify open questions and test our speculations. For example, further studies could localize EBA, MT, MST, and the “action-related region” (Astafiev et al., 2004; Peelen & Downing, 2005b) elicited by unseen pointing movements. Within participants, activity in these regions could be measured during observation of hand movements, as here; during control of visual cursors, as in David et al. (2007); during passive somatosensory stimulation; during visually guided, but unseen movements (Astafiev et al., 2004); and during a spatial attention task (Gitelman et al., 1999).

Aside from the studies by David and colleagues reviewed above, the question of whether EBA and FBA distinguish the self from others has been addressed primarily with static images, in which the cues to self/other identity are pictorial rather than motoric. Two studies revealed that right, but not left, EBA showed a slight preference for allocentric over egocentric views of bodies (Chan, Peelen, & Downing, 2004) and body parts (Saxe, Jamal, & Powell, 2006), but no difference was found between images of the bodies of the self and others (Chan et al., 2004). This is in contrast to the results of Myers and Sowden (2008), who used an fMRI adaptation technique to show sensitivity in EBA to this distinction, using hands as stimuli. These studies did not consider FBA, but more recently two studies (Hodzic, Kaas, Muckli, Stirn, & Singer, 2009; Hodzic, Muckli, Singer, & Stirn, 2009) indicate that FBA distinguishes whole-body images of the self from others – and may more generally represent the identity of individuals – in contrast to EBA, which showed no such sensitivities (see also Peelen & Downing, 2007; Taylor, Wiggett, & Downing, 2007).

In the present study, in contrast to EBA and FBA, a region of the posterior STS showed modulation of the response to visual images of moving hands by concurrent motor output. The response to the Incompatible condition was greater than to the Compatible condition. Strikingly, the Compatible condition was not significantly different from the Do condition—in other words, the net response of this region to a live view of one's own actions is similar to that elicited when no visual stimulus is presented at all.

What is the source of the modulation found in pSTS, and why does it occur? One possibility is that the representation of expected biological stimuli is suppressed in pSTS—e.g. by means of an efferent copy generated elsewhere. This is the model that Leube et al. (2003) proposed for their findings (described in the Introduction), implicating the cerebellum and the putamen in the generation of the forward model that anticipates sensory consequences of movements. (To explain the present results [specifically the finding that Watch > Incompatible] would also require assuming a general suppression of pSTS during motoric activity, which is also compatible with the below-baseline response of this region during the “Do” condition.) However, we did not identify cerebellum or putamen in our whole-brain contrasts of Incompatible vs Compatible.

In the Leube et al. (2003) task, the error between the perceived and generated hand movements was subtle, and took the form of a small timing lag in the video loop. In contrast, in the present study, in the Incompatible condition the perceived and generated movements were grossly incompatible, in that the series of actions was performed in a different order. In that sense they seem less likely to engage cerebellar error signals, and rather cognitive error or conflict signals, elicited by the symbolic mismatch between the executed action series (e.g. ABC) and the viewed series (e.g. CBA). Indeed, in the whole-brain Incompatible vs Compatible contrast, we identified increased activity in the dorsal anterior cingulate/pre-SMA region, consistent with previous findings implicating this area in cognitive conflict—for example in the Stroop task (Carter et al., 2000). We also identified activations of the right temporal parietal junction (dorsal to our pSTS ROI), a region that is regularly found to respond to unexpected perceptual events, particularly when they evoke a shift of attention (Shulman et al., 2009). Hence we speculate that the relative suppression in pSTS in the Compatible, relative to the Incompatible, condition might be better attributed to (in)congruency between the visual and motor systems at a cognitive level, rather than sensory attenuation by the outputs of a forward model.

Both of the above scenarios deal with the relatively lower response in pSTS to Compatible relative to Incompatible conditions. In contrast, Iacoboni et al. (1999) reported an *increase* in pSTS activity when participants viewed hand movements while also moving their own hands in an effort to imitate these, compared to the visual stimulus alone. This pattern is the opposite of what was found here. In Iacoboni et al.’s (1999) study, the task required imitation, whereas here the video stimuli either matched or mismatched participants’ actions, but no imitation was required. pSTS coding of actions may be flexible, such that visual representations are subject to modulation by motor activity, contingent on the requirements of the task.

In addition to the stimulus/task modulation of pSTS responses, the timecourse data also show a rise in the BOLD response in this region early in the experimental blocks, and again immediately following the end of these blocks. These appear to be driven by the task cues and the end of the blocks, respectively, and may reflect a general sensitivity to transitions in visual stimulation and/or motor activity.

The overall pattern of results in this study can rule out several potential confounds. One possibility is that participants may attend to the visual input to different degrees depending on the concurrent motor task. For example, it could, in principle, have been the case that visual attention to the moving hands was decreased (or indeed increased) in the Incompatible condition, compared to the Compatible condition, because of the conflict in the former case between the images and the required movements. However, the finding that EBA and FBA responded to these conditions equally, whereas pSTS did not, rules out any account based on global differences in attention to the stimuli (assuming activity in the former areas is modulated by attention as in other similar extrastriate areas; cf Wojciulik, Kanwisher, & Driver, 1998). This dissociation, along with the whole-brain results showing significant activation in other areas to Incompatible vs Compatible conditions, also argues against the possibility that the absence of a difference in EBA and FBA was due to insufficient power. More generally, the fronto-parietal pattern seen in the whole-brain contrast is expected given the relative difficulty of the Incompatible condition relative to the Compatible condition (Duncan, 2006). This makes it all the more surprising, in our view, that EBA/FBA activity is not modulated accordingly, e.g. via feedback connections. Finally, potential order effects must be considered, because the sequence of conditions was constant in each scan. A given video action sequence was seen three times in a given scan—first when it was viewed (and recorded) during the Compatible condition; again in the following Incompatible condition; and finally in the Watch condition. Normally, repetition of stimuli reduces BOLD responses in extrastriate visual areas (e.g. Vuilleumier, Henson, Driver, & Dolan, 2002), whereas in pSTS (but not EBA and FBA), we see trends towards increasing responses across these repetitions, so adaptation is unlikely to account for the effects seen here.

## Figures and Tables

**Table 1:** All activations for which (1) Incompatible > Compatible or (2) Compatible > Incompatible, from a whole-brain, random-effects analysis, thresholded at *t* = 3.58, *p* < 0.005, cluster-size threshold: 6 voxels.

Region	Extent (mm^3^)	Mean peak			Max(*t*)
		*X*	*Y*	*Z*	
*Incompatible > Compatible*					
R superior temporal sulcus/gyrus	1915	57	−55	19	7.8
R middle temporal gyrus	1247	60	−46	−5	9.59
R middle frontal gyrus	396	45	12	22	6.65
R inferior frontal gyrus	312	48	17	2	6.04
R Precuneus	275	9	−55	34	6.2
R middle frontal gyrus	246	42	5	34	6.17
Superior frontal gyrus	1873	0	38	43	6.94
L superior temporal sulcus/gyrus	7020	−54	−52	16	8.81
L superior frontal gyrus	419	−6	8	55	5.22
L middle frontal gyrus	290	−45	11	31	6.21
L middle temporal gyrus	206	−42	−46	−8	8.86
L inferior frontal gyrus	192	−42	20	2	6.1

*Compatible > Incompatible*					
R parieto-occipital sulcus	688	24	−85	1	9.21
R parieto-occipital sulcus	364	24	−76	16	5.72

Each row gives the location of the cluster, the volume of the activation, the location of the peak voxel of that activation in Talairach co-ordinates, and the maximum *t* value for the region.

## Footnotes

1 Although David et al. (2007) reported a significant effect of congruence between action and visual stimulus in the right but not left EBA, David et al. (2009) stimulated left EBA with their TMS procedure. To examine whether congruence effects were present in our left hemisphere data, we examined left EBA (mean Talairach coordinates of peaks: *x* = −47.8 (1.55), *y* = −66.6 (1.82), *z* = 1 (2.9)). The results were similar to those found for right EBA: Compatible mean = 6.70 (SE = 0.85); Incompatible 7.30 (0.89); Watch 7.26 (1.02); Do 3.80 (0.94). Critically, there was no significant difference between the Compatible and Incompatible conditions in left EBA, *t*(10) = 1.66, *p* = 0.13.
